# Do Bacteremic patients with end-stage renal disease have a fever when presenting to the emergency department? A paired, retrospective cohort study

**DOI:** 10.1186/s12873-019-0298-2

**Published:** 2020-01-09

**Authors:** Sarah L. Weatherall, Alison B. Chambers, Leonard A. Mermel

**Affiliations:** 1 Warren Alpert Medical School of Brown University, Providence, RI USA; 2 Department of Medicine, Warren Alpert Medical School of Brown University, Providence, RI USA; 3 Lifespan Biostatistics Core, Rhode Island Hospital, Providence, RI USA; 4 Department of Medicine, Warren Alpert Medical School of Brown University, Providence, RI USA; 5 Division if Infectious Diseases, Rhode Island Hospital, 593 Eddy St, Providence, RI 02903 USA

**Keywords:** *Staphylococcus aureus*, Bacteremia, Hemodialysis, End-stage renal disease, Fever

## Abstract

**Background:**

Fever is a common symptom when patients present to Emergency Departments. It is unclear if the febrile response of bacteremic hemodialysis-dependent patients differs from bacteremic patients not receiving hemodialysis. The objective of this study was to compare Emergency Departments triage temperatures of patients with and without hemodialysis-dependent end-stage rental disease who have *Staphylococcus aureus* bacteremia and determine the incidence of afebrile *S. aureus* bacteremia.

**Methods:**

Paired, retrospective cohort study of 37 patients with and 37 patients without hemodialysis hospitalized with Methicillin-resistant or Methicillin-susceptible *S. aureus* bacteremia. Emergency Department triage temperatures were reviewed for all patients, as were potential confounding variables.

**Results:**

54% (95% CI, 38–70%) and 82% (95% CI 65–91%) of hemodialysis and non-hemodialysis patients did not have a detectable fever (<100.4 °F) at triage. Triage temperatures were 100.5 °F (95% CI 99.9–101.2 °F) and 99.0 °F (95% CI 98.4–99.6 °F) in the hemodialysis and non-hemodialysis cohorts, respectively (*p* < 0.001). Triage temperature in patients with and without diabetes mellitus was 99.2 °F (95% CI 98.4–99.9 °F) and 100.4 °F (95% CI 99.7–101.0 °F), respectively (*p* = 0.03). We were unable to detect a significant effect of diabetes mellitus and other potential confounding variables on differences in temperature between the hemodialysis and non-hemodialysis cohorts (all interactions *p* > 0.19).

**Conclusions:**

Hemodialysis-dependent patients with *S. aureus* bacteremia had significantly higher temperatures than non- hemodialysis-dependent end stage renal disease patients but more than half of patients were without detectable fever at triage, possibly reflecting use of insensitive methods for measuring temperature. Absence of fever at presentation to the Emergency Department should not delay blood culture acquisition in patients who are at increased risk of *S. aureus* bacteremia.

## Background

Studies examining the febrile response of infected patients with hemodialysis-dependent end-stage renal disease (ESRD) are scarce, and the literature on hemodialysis patients’ baseline body temperatures is contradictory. There is some evidence that hemodialysis patients have low basal body temperatures [1, 2], suggesting that their maximal temperatures during active infection may be less than those of other patient populations. Bacteremic patients with reduced renal function have been found to have a blunted febrile response compared to those with preserved renal function; however, patients receiving dialysis and/or having “rapid fluctuation of serum creatinine levels” were notably omitted from the study [3]. More recent evidence suggests that hemodialysis patients have higher basal temperatures compared to healthy controls [4], raising the possibility of dialyzed patients reaching higher maximum temperatures during episodes of infection than their non-dialyzed counterparts.

## Methods

The primary aim of this study was to compare the body temperatures of patients with and without hemodialysis-dependent ESRD who had proven *Staphylococcus aureus* bacteremia, in attempt to compare the presence of fever in those patients. We chose to study patients with blood cultures growing *S. aureus* because these are rarely considered contaminants (e.g., only 1% of cultures growing *S. aureus* are considered contaminants [5], and *S. aureus* is a particularly common cause of bacteremia, especially among hemodialysis patients [6, 7]). An additional goal of this study was to determine the frequency of patients with afebrile *S. aureus* bacteremia, as absence of fever has been associated with diagnostic delay and poor health outcomes [8, 9].

We conducted a paired, retrospective cohort study of patients with *S. aureus* bacteremia at Rhode Island Hospital (RIH) between January 1, 2015 and December 31, 2017. RIH is a tertiary care referral center licensed for 719-beds, with approximately 150,000 annual Emergency Department (ED) visits. All patients with blood cultures that grew *S. aureus* at RIH during the study period were identified using in the RIH Infection Control Department software program (Theradoc, Premier, Charlotte, NC). We determined which of these patients were on chronic hemodialysis (HD cohort) by reviewing the medical record. We separated these patients from the remainder and used the random number generator function in Excel to assign each of the remaining non-hemodialysis patients (No HD cohort) a random identification number. We then sorted this group by the random identification number. We included the first person on the randomly-sorted No HD list in the study if their sex and age (within 10 years) matched that of the person on the HD list. If the sex and age did not match, we included the next person on the randomly sorted list, and so on. This study was approved by the RIH Institutional Review Board with a waiver of informed consent.

The electronic health record (Epic, Verona, WI) was reviewed for each case of *S. aureus* bacteremia to identify patients with hemodialysis-dependent ESRD. A comparison cohort of patients without hemodialysis-dependent ESRD were selected from the remaining cases of *S. aureus* bacteremia using a random number generator. The hemodialysis cohort and the comparison cohort were matched for sex and age (within 10 years), as both sex and age have been shown to be associated with a febrile response to infection [10, 11]. Patients ≥18 years of age were included if at least one percutaneously-drawn blood culture obtained within 48 h of ED presentation grew Methicillin-sensitive *S. aureus* (MSSA) or Methicillin-resistant *S. aureus* (MRSA). Patients were excluded if they were on peritoneal dialysis or taking systemic steroids prior to presentation. If patients had more than one episode of MSSA or MRSA bacteremia during the study period, then only the first episode was included.

The ED provider notes, admission history and physical exam, Infectious Diseases consult note, Emergency Medical Services run sheet, skilled nursing facility records, and hemodialysis records were reviewed. If patients were transferred to RIH from an outside hospital, then the outside records were reviewed, and data from the initial ED visit were included in the analysis. The data collected included initial temperature recorded in the ED (e.g., triage temperature), age, sex, chief complaint of fever and/or chills, and blood culture results. We also assessed variables thought to directly affect patient temperature, including thermometer type (e.g., tympanic membrane, rectal), antipyretic use prior to ED presentation (e.g., acetaminophen, non-steroidal anti-inflammatory medications), antibiotic use prior to ED presentation, and medical conditions with potential temperature-altering effects (e.g., hypothyroidism, cirrhosis). Additionally, patient location prior to ED presentation (e.g., home, skilled nursing facility, dialysis unit) was collected as a proxy of premorbid condition and level of access to medical evaluation. Medical conditions common in the ESRD population were also recorded (e.g., diabetes mellitus, congestive heart failure, cerebrovascular disease). Lastly, to estimate hemodialysis patients’ baseline temperatures prior to mounting a possible febrile response, we obtained their pre-dialysis temperature at the most recent dialysis session prior to ED presentation.

A general estimation equation (normal distribution) was used to model temperature in each cohort (HD or non-hemodialysis group). An interaction term was added to the main model to test the effect of confounding variables on temperature difference between the HD and No HD group. Confounders for patient sickness (presenting from home or from other facility), direct temperature effects (antipyretic use, hypothyroidism, cirrhosis) and common chronic kidney disease comorbidities (diabetes mellitus, congestive heart failure, cerebrovascular disease) were tested. The rate of patients reporting fever and/or chills as a chief complaint (chills or no chills) was also modeled using a generalized estimating equation (logistic) by hemodialysis group, with an interaction term for patients’ ED temperature (allowing for differing affects with increasing ED temperature). Nesting for gender and age matched pairs was accounted for in all models.

Additionally, temperature of HD patients during their most recent HD treatment was compared to their ED temperature and compared to the absolute value of 98.6 °F.

Sandwich estimation was used to adjust for model misspecification for all models run. In all cases, statistical significance was set to *p* < 0.05 a priori. SAS was used for all statistical analyses (version 9.4, Cary, NC).

## Results

During the study period, there were 428 patients with blood cultures that grew MSSA or MRSA. 48 (11%) of these patients were on chronic hemodialysis for ESRD at the time of presentation. Eleven hemodialysis-dependent patients were excluded from the study: four with positive blood cultures drawn only from an intravascular catheter (e.g., no positive percutaneously-drawn cultures); two with positive blood cultures drawn more than 48 h after admission; three with documented oral steroid use prior to presentation; and two with incomplete ED records.

Thirty-seven patients each in the HD and No HD cohorts were included in the study. 70% of patients were male; mean age was 63 years in both cohorts. A temporal artery thermometer was the predominant method of thermometry; one patient was known to have their temperature monitored centrally (i.e., rectal thermometer; Table 1).

**Table 1:** Baseline characteristics of cohorts

	Hemodialysis Patients *n* = 37 (%)	Non-Hemodialysis Patients *n* = 37 (%)
Age, mean (SD)	63 (17)	63 (17)
Sex		
Female	11 (30)	11 (30)
Male	26 (70)	26 (70)
Comorbidities		
Hypothyroidism	3 (8.1)	4 (11)
Cirrhosis	2 (5.4)	3 (8.1)
Congestive Heart Failure	15 (41)	7 (19)
Diabetes mellitus	21 (57)	13 (35)
Cerebrovascular Disease	5 (14)	6 (16)
Presented From		
Home	17 (46)	29 (78)
Nursing Facility	12 (32)	6 (16)
Dialysis Unit	7 (19)	0 (0.0)
Outpatient Clinic/ Urgent Care	1 (2.7)	2 (5.4)
Antipyretic Use Prior to Triage	10 (27)	8 (22)
Antimicrobial Use Prior to Triage		
Use of any antimicrobial	5 (14)	2 (5.4)
Use of effective antimicrobial^1^	2 (5.4)	0 (0.0)
Chief Complaint of Fever/Chills	22 (59)	14 (38)
Thermometer Type Used at Triage		
Temporal artery	21 (57)	25 (68)
Oral	4 (11)	4 (11)
Rectal	1 (2.7)	0 (0.0)
Tympanic membrane	1 (2.7)	0 (0.0)
Unknown	10 (27)	8 (22)

^1^Effective antimicrobial therapy was defined as Beta-lactam antibiotic or first- generation cephalosporin for MSSA and Vancomycin or Daptomycin for MRSA bacteremia

**Fig. 1:**
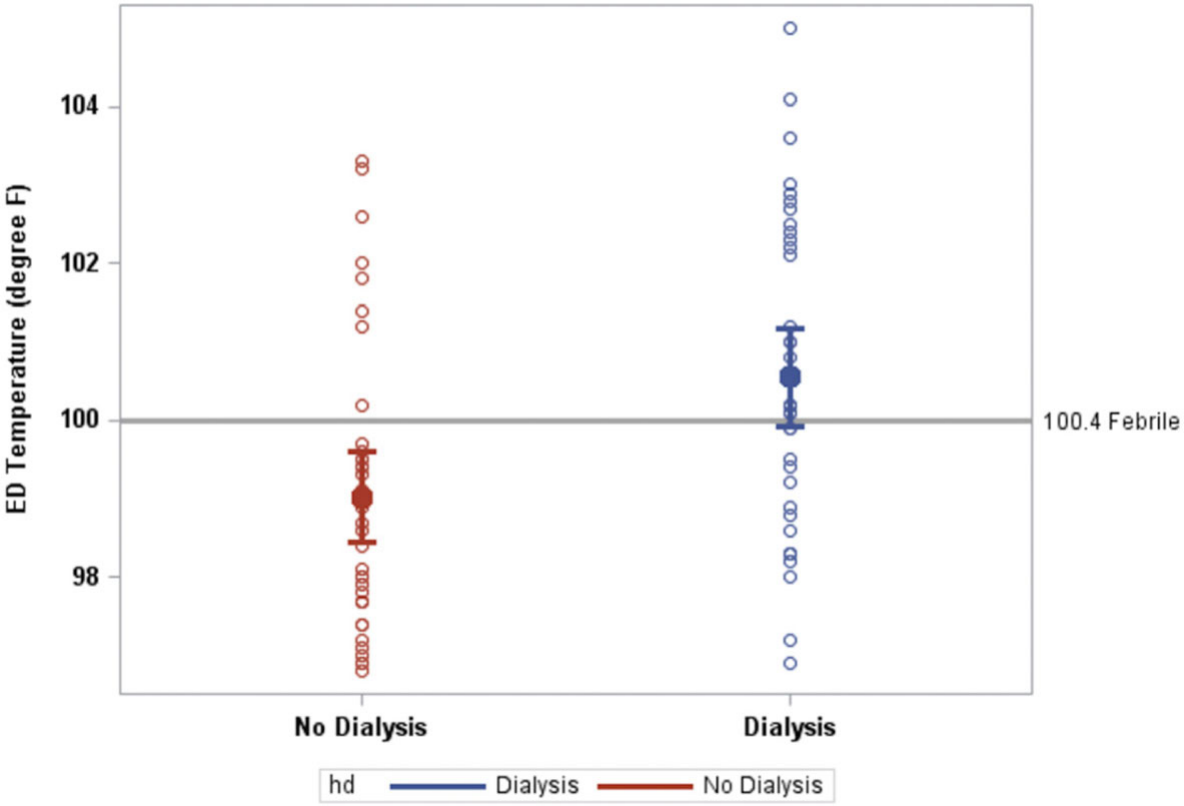
ED triage temperature of HD patients and No HD patients (*p* < 0.001). Filled circles represent group means; unfilled circles represent individual observations. Bars indicate 95% CI. Gray reference line represents 100.4 °F as a threshold for febrile

**Fig. 2:**
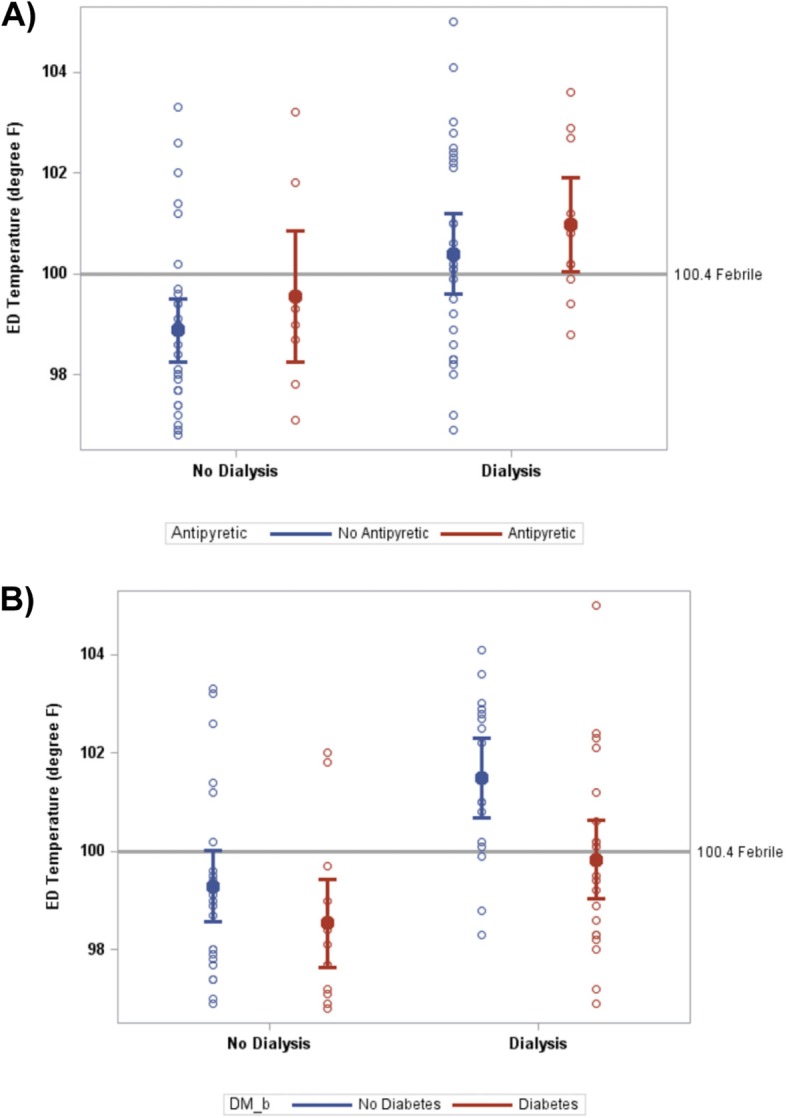
ED triage temperature of HD patients versus No HD patients by A) level of antipyretic and B) level of diabetes. Filled circles represent group means; unfilled circles represent individual observations. Bars indicate 95% ci. Gray reference line represents 100.4 °F as a threshold for febrile

54% (95% CI, 38–70%) and 82% (95% CI, 65–91%) of hemodialysis and non-hemodialysis patients were without detectable fever at triage (i.e., temperature < 100.4 °F). Estimated mean ED triage temperatures were 100.5 °F ([95% CI 99.9–101.2 °F) and 99.0 °F (95% CI 98.4–99.6 °F) in the HD and No HD cohorts, respectively (*p* < 0.001; Fig. 1). Estimated mean ED triage temperature in patients who had and had not received antipyretics prior to the ED visit was 100.3 °F (95% CI, 99.4–101.2 °F and 99.6 °F (95% CI, 99.1–100.2 °F) respectively, (*p* = 0.17; Fig. 2a). Estimated mean ED triage temperature in patients with and without diabetes mellitus was 99.2 °F (95% CI, 98.4–99.9 °F) and 100.4 °F (95% CI, 99.7–101.0 °F), respectively (*p* = 0.025; Fig. 2b). We were unable to detect a significant effect of antipyretic use prior to presentation and a diagnosis of diabetes mellitus on differences in temperature between the HD and No HD cohorts (both interactions *p* > 0.19). Temperature differences between the HD and No HD cohorts were maintained considering other comorbidities or factors indicative of patients’ premorbid condition (all interactions *p* > 0.19; Table 2).

**Table 2:** Effect of potential confounding variables on temperature differences between hemodialysis (HD) and non-hemodialysis (no HD) cohorts

Variable	HD Temperature °F (95% CI)	No HD Temperature °F (95% CI)	*P*-value	Interaction *p*-value
Antipyretic	101.0 (100.0–102.0)	99.6 (98.2–100.9)	0.33	0.93
No antipyretic	100.4 (99.6–101.2)	98.9 (98.2–99.5)	0.04	
Hypothyroidism	101.2 (99.3–103.1)	99.5 (97.1–101.9)	0.67	0.92
No hypothyroidism	100.5 (99.8–101.2)	99.0 (98.4–99.6)	0.02	
Cirrhosis	99.6 (98.6–100.5)	98.1 (97.1–99.2)	0.18	0.91
No cirrhosis	100.6 (99.9–101.3)	99.1 (98.5–99.7)	0.01	
Diabetes mellitus	99.8 (99.0–100.7)	98.5 (97.6–99.5)	0.05	0.20
No diabetes mellitus	101.5 (100.6–102.3)	99.3 (98.5–100.1)	<0.001	
Congestive heart failure	100.4 (99.5–101.4)	98.19 (96.8–99.6)	0.05	0.40
No congestive heart failure	100.7 (99.8–101.6)	99.2 (98.6–99.9)	0.03	
Cerebrovascular disease	99.6 (98.1–101.1)	98.4 (97.1–99.6)	0.53	0.75
No cerebrovascular disease	100.7 (100.0–101.4)	99.2 (98.5–99.8)	0.01	
Presented from home	100.8 (99.8–101.8)	99.1 (98.4–99.9)	0.05	0.91
Did not present from home	100.4 (99.5–101.2)	98.6 (97.7–99.5)	0.03	

**Fig. 3:**
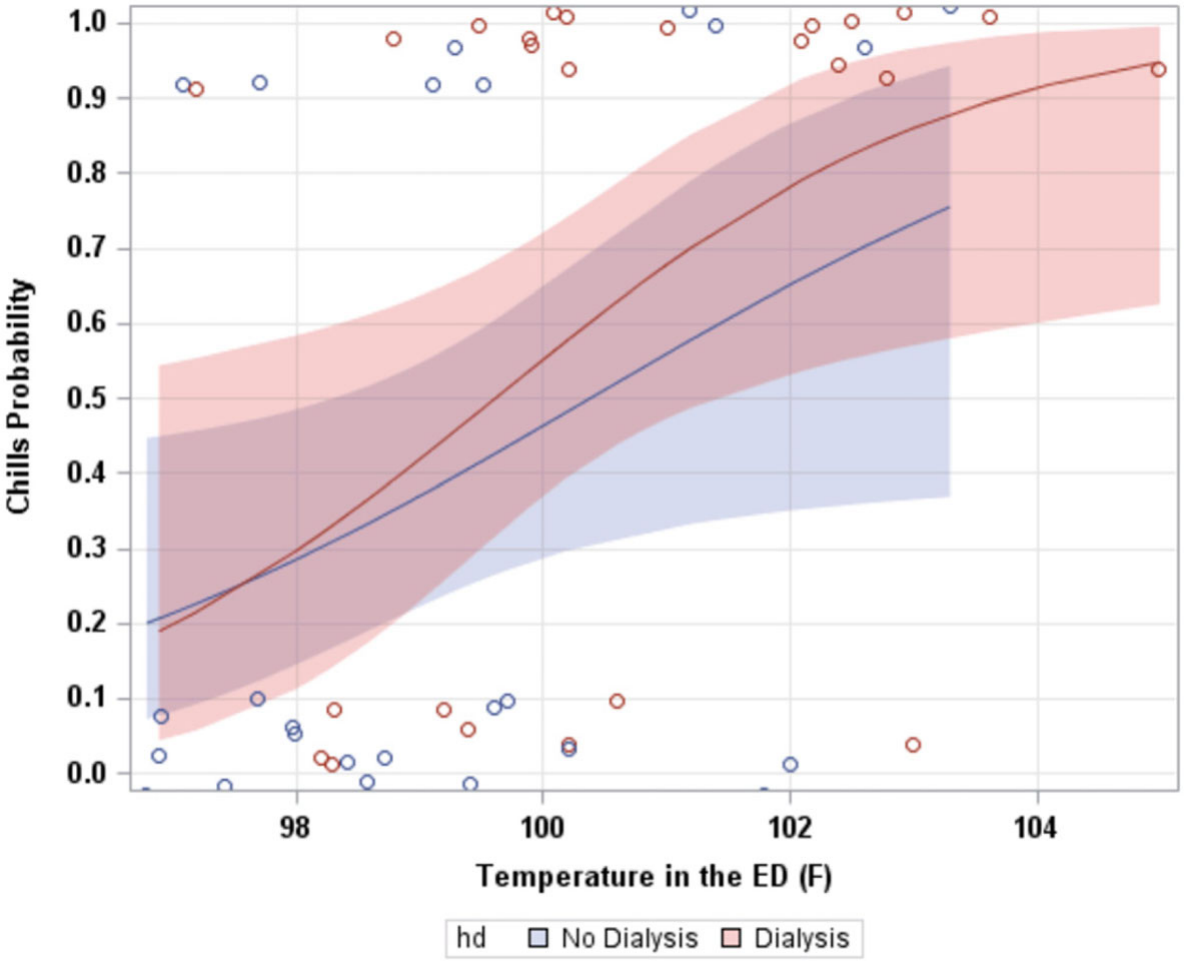
Blue (No HD) and red (HD) lines represent probability of a patient reporting fever and/or chills with increasing ED triage temperature by level of dialysis. Blue and red shaded regions represent 95% confidence intervals

Increasing ED triage temperature was associated with higher likelihood of patient-reported fever and/or chills (*p* = 0.006). Whether a patient was HD or No HD did not impact the likelihood of a chief complaint of fever and/or chills with increasing ED triage temperature (interaction *p* = 0.595, Fig. 3).

**Fig. 4:**
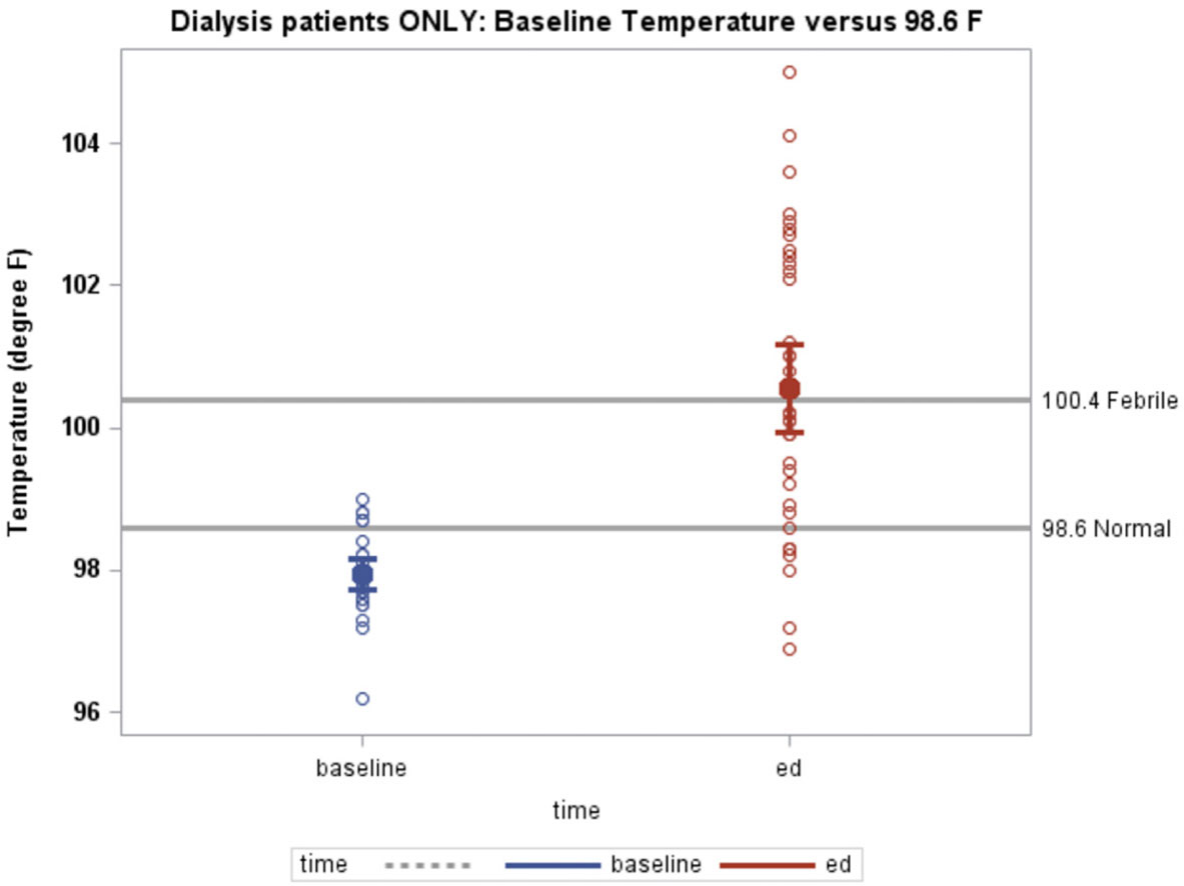
HD patients’ temperature at baseline compared to ED triage temperature. Baseline temperature versus 98.6 °F. Gray reference lines represent 100.4 °F as a threshold for febrile and 98.6 °F as a threshold for normal temperature

At baseline, HD patients had an estimated mean temperature of 98.0 °F (95% CI 97.7–98.2 °F), lower than 98.6 °F (*p* < 0.001). HD patients had higher ED triage temperatures compared to their baseline temperatures (100.6 °F [95% CI 99.9–101.2 °F] and 98.0 °F [95% CI 97.7–98.2 °F], respectively, *p* < 0.001, Fig. 4).

## Discussion

Given the scant literature regarding the effect of hemodialysis on the febrile response to infection, we compared the body temperatures of patients with and without hemodialysis-dependent ESRD who had proven *S. aureus* bacteremia in attempt to clarify differences in presence of fever. HD patients had higher temperatures compared to patients in the comparison cohort (*p* < 0.001). The reason for higher temperatures in HD patients is unclear. It is possible that hemodialysis raises basal body temperatures, leading to higher temperatures among hemodialysis patients during episodes of infection. Some authors speculate that chronic inflammation may play a role [4]. Chronic inflammation may be due to repeated exposure to dialysate and dialysis water contaminated with gram-negative bacteria [12]. This results in release of endotoxin through permeable hemodialysis membranes [13], leading to production of proinflammatory cytokines. Endotoxin (e.g. lipopolysaccharide, LPS) and proinflammatory cytokines are exogenous and endogenous pyrogens, respectively, and both raise core body temperature [14]. Another possible explanation for hemodialysis patients’ elevated temperature is the hemodynamic response to dialysis. During hemodialysis, increased metabolic rate and peripheral vasoconstriction lead to increased heat production and decreased heat loss, respectively. Unless the dialysate temperature is reduced, these hemodynamics result in increased body temperature [2].

We assessed the potential impact of several possible confounding variables on the temperature differences observed between the HD and No HD cohorts. We were particularly concerned about the influence of antipyretic use [15], but we were unable to detect a significant effect of antipyretic use on differences in temperature between the HD and No HD cohorts. Interestingly, patients who took antipyretics prior to ED presentation had higher temperatures at triage compared to those who did not, although this difference was not significant. This suggests that patients with fever prior to hospitalization may have been more likely to consume antipyretics for symptomatic relief. In fact, a chief complaint of fever and/or chills was associated with increased temperature at triage (*p* = 0.01).

We also tested the impact of several medical comorbidities common in ESRD patients and/or known to affect body temperature. Diabetes mellitus was shown to have a significant effect on body temperature, with diabetic patients having significantly lower temperatures compared to non-diabetic patients. This finding may be explained by impaired thermoregulation in this population secondary to a number of factors such as autonomic dysfunction [16]. Nevertheless, we did not have the ability to detect a significant effect of diabetes mellitus on temperature differences between the HD and No HD cohorts (interaction *p*-value 0.20).

A secondary aim of our study was to determine the percentage of patients with afebrile bacteremia. Surprisingly, we found that over half of HD and No HD patients did not have detectable fever (temperature > 100.4 °F) when initially assessed at ED triage, despite concurrent *S. aureus* bacteremia. The large proportion of afebrile patients in our study is concerning, as fever is often the first clue in the diagnosis of bacteremia [17], and the impetus for physicians to order blood cultures and initiate antimicrobial therapy. According to the Surviving Sepsis Campaign guidelines [18], empiric broad-spectrum antibiotics should be started immediately in patients suspected of having sepsis after blood cultures have been obtained. Each hour delay in antibiotic administration is associated with increased mortality [19]. It is not surprising that afebrile bacteremia is associated with worse outcomes [9], as septic patients presenting without fever are more likely to experience diagnostic and treatment delays.

A concern raised by the high proportion of afebrile patients in our study is whether we are reliably detecting fever in infected patients when they initially present to the ED. Of the patients for whom a thermometer type was documented in the medical record, peripheral thermometers (e.g. temporal artery, oral, tympanic membrane) were used to measure temperature in all but one patient (Table 1). When compared to central thermometry, peripheral thermometers are inaccurate, with poor sensitivity for detecting fever [20]. A temporal artery thermometer was the most common thermometer used in our study and is among the least accurate of the peripheral thermometer types. Pulmonary artery, urinary bladder catheter, esophageal, and rectal thermometers are the most accurate methods of measuring temperature [21]. While invasive, these central thermometers detect fever more reliably in patients presenting with life-threatening infections (e.g. *S. aureus* bacteremia), and likely lead to reduced diagnostic delay and possibly reduced mortality.

Our study has several limitations. Our analysis of patients’ temperature was limited to their assessment at ED triage. It is likely that patients’ temperatures fluctuate throughout hospitalization, and as such, other studies have looked at the presence of fever throughout the ED course [9] or 24 h prior to blood culture acquisition [10]. Nonetheless, ED triage serves as the first point of contact in the hospital setting, and the high prevalence of bacteremic patients at triage without detection of fever raises important concerns, given the association between afebrile bacteremia and diagnostic delay [8]. Due to the retrospective nature of the study, we were unable to ensure that the same thermometer was used to measure temperature in all patients, and we had no way of confirming that the thermometers were properly calibrated. While these inconsistencies pose experimental challenges, they are reflective of a true clinical scenario in the ED. Furthermore, we did not check central temperatures, so we are unable to confirm our hypothesis that in some patients, fever was undetected due to use of temporal artery thermometry. We did not test the effect of race or diurnal variation on temperature differences between the hemodialysis and comparison cohorts either. Both race [22] and time of day [11] have been shown to significantly affect body temperature in other studies. Lastly, because our sample size was small, our study was not powered to detect differences in the covariates.

## Conclusion

Bacteremic hemodialysis patients had significantly higher body temperatures than those without hemodialysis-dependent ESRD. We were unable to detect differences caused by antipyretic use, medical conditions known to affect temperature, common comorbidities in patients with ESRD, or presentation to the ED from home. The reason for this temperature difference is unclear, but may be related to changes in baseline body temperature secondary to the effects of hemodialysis. Furthermore, our study showed that a surprisingly large percentage of HD and No HD patients were without detectable fever at ED triage. This finding may reflect use of insensitive methods for measuring body temperature, given the frequent use of peripheral thermometers. Central thermometers, which have greater sensitivity for detecting fever, should be considered when triaging patients at high risk of *S. aureus* bacteremia (e.g. hemodialysis patients). Absence of detectable fever should not be a reason to delay blood culture acquisition or empiric treatment in patients who are at increased risk for *S. aureus* bacteremia and in whom a serious infection is on the differential diagnosis.

## Data Availability

The datasets used and/or analysed during the current study are available from the corresponding author on reasonable request.

## Abbreviations

ED: Emergency Department
ESRD: Hemodialysis-dependent end-stage renal disease
HD: Hemodialysis group
MRSA: Methicillin-resistant *S. aureus*
MSSA: Methicillin-sensitive *S. aureus*
No HD: Non-hemodialysis group
RIH: Rhode Island Hospital
